# Peroxidase Sensor Based on Electroactive Ce^3+^-Phytic Acid-Cu^2+^@Cu Composite

**DOI:** 10.3390/molecules31193448

**Published:** 2026-09-28

**Authors:** Boxin Dou, Minrong Li, Mingyu Li, Yan Wang, Jianhui Jia, Ying Liu

**Affiliations:** 1 Key Laboratory of Food Science and Engineering of Heilongjiang Ordinary Higher Colleges, College of Food Engineering, Harbin University of Commerce, Harbin 150000, China; lmr172265@163.com (M.L.); mingxiaoyua@163.com (M.L.); wangyan_123456@163.com (Y.W.); liu15114669883@163.com (Y.L.); 2 Postdoctoral Research Workstation of Northeast Asia Service Outsourcing Research Center, Harbin University of Commerce, Harbin 150028, China; 3 College of Life Science and Technology, Mudanjiang Normal University, Mudanjiang 157011, China; jiajianhui@yeah.net

**Keywords:** cerium-based nanocomposite, H_2_O_2_ detection, electrochemical sensor

## Abstract

Peroxide is a vital oxidant that can quickly kill bacteria, viruses, and fungi in food and ensure food safety. However, excessive intake will stimulate the gastrointestinal tract, cause abdominal pain and vomiting, and its strong oxidation will also destroy the vitamin and protein structure in food. Cerium-based nanomaterials exhibit good biocompatibility and strong chemical stability, and have significant application value in biological detection. Cu nanoparticles were deposited on the surface of a glassy carbon electrode (GCE) by potentiostatic deposition. The Ce^3+^/PA-Cu^2+^@Cu/GCE sensor was constructed by etching and complexing Cu^2+^ with phytic acid (PA) to form a PA-Cu^3+^ coordination film. By X-ray diffraction (XRD) and Fourier Transform Infrared (FT-IR), the structure of composite material on the electrode surface was characterized. Electrochemical test results: Cu time of electrodeposition 700 s, PA embellishment 60 min, Ce^3+^ coordination 60 min, and H_2_O_2_ Reaction 15 min. The detection linearity range of the sensor for hydrogen peroxide is 0.1–100 μmol/L, detection limit is 0.89 μmol/L, and there was good selectivity, sensitivity and reproducibility (RSD = 1.013%) and stability (retention of 84% response at 28 days).

## 1. Introduction

As a class of chemicals with strong oxidizing properties, peroxides are widely used as disinfectants and preservatives in the field of food processing. They extend the shelf life of packaged food by inhibiting the metabolic activities of microorganisms. However, excessive use of this substance may bring many risks. In food production, the illegal addition of excessive peroxide will not only lead to the decomposition of vitamins, proteins and other nutrients, but also damage the digestive tract mucosa, causing abdominal pain and vomiting [1]. Therefore, the detection of peroxide is of great significance to protecting food safety, environmental health and industrial production safety. Common detection methods include titration, chromatography, fluorescence, chemiluminescence, spectrophotometry and electrochemical methods. Among them, electrochemical detection, as an analytical technology, has the basic characteristics of fast response, high sensitivity and easy operation, and has received wide attention. Comparing the advantages and disadvantages of different methods, it can be seen that the electrochemical method has the best comprehensive performance. Therefore, this paper identifies the electrochemical method as the main research method for manufacturing various H_2_O_2_ detection electrodes.

Electrochemical biosensors mainly use the specific three-dimensional structure of biological small molecules and their specific identification ability of the corresponding substrates to convert the concentration-related parameters of the target object to be tested into electrical signal parameters, and then record the generated signals and process them [2,3,4]. The specificity of biomolecular identification determines the high selectivity of electrochemical biosensors, which have the advantages of speed, strong specificity, sensitivity and easy miniaturization. It has attracted wide attention from researchers in the analysis and detection of peroxide [5,6,7,8]. The surface of the biosensor needs to modify enzyme-active substances or construct a nanofilm structure with simulated enzyme activity. The simulated enzyme materials used to build H_2_O_2_ electrochemical sensors include precious metals, metal oxides, carbon nanomaterials and metal organic frameworks (MOFs), etc. Compared with natural enzyme biosensors, electrochemical sensors based on analog enzymes and their composites can amplify the sensing signal, reduce the redox potential of H_2_O_2_, improve sensitivity and enhance stability. A copper-based metal organic skeleton (mof) has the advantages of rich metal potential, large specific surface area, simple synthesis process, etc., and is a promising sensing material. Yong Li [9] prepared silver-doped copper oxide nanocomposites (Ag@CuO) by electrostatic spinning method, and obtained nanoparticles with honeycomb structure through heat treatment. Material characterization shows that the nanocomposite has high purity and excellent electrocatalytic properties. Experiments show that compared with pure CuO modified electrodes, the voltammetry response of Ag@CuO-modified carbon slurry electrodes (CPE) is 3 times better, and it has significant catalytic activity for H_2_O_2_ oxidation. The detection performance of the sensor reaches sensitivity 1982.14 μA (mmol·L^−1^), detection limit 0.01 μmol·L^−1^ (S/N = 3), and a linear coverage of 0.05 μmol· L^−1^ to 1.5 mmol L^−1^. This method was successfully used to determine H_2_O_2_ in canned coconuts, with a recovery rate of 98.7% to 102.3%, verifying its practical application value. Cerium-based materials (such as CeO_2_ and Ce-MOFs) have abundant oxygen vacancy, strong Ce^3+^/Ce^4+^ valence conversion ability, and high oxygen storage capacity. They can simulate peroxidase activity, accelerate H_2_O_2_ decomposition reaction, and improve electrocatalytic performance. Manibalan G et al. [10] developed silver (Ag)-doped CeO_2_/Ag_2_O-modified glass carbon electrode (Ag-CeO_2_/Ag_2_O/GCE) as a non-enzymatic electrochemical sensor, which is used for sensitive and selective detection of H_2_O_2_ to show excellent electrocatalytic activity. This is due to the increase in active sites and the enhancement of electron transfer. The sensor has a high sensitivity of 2.728 µA cm^−2^µM^−1^, which is significantly better than the undoped CeO_2_/GCE (0.0404 µA cm^−2^µM^−1^). In the wide linear detection range of 1 × 10^−8^~0.5 × 10^−3^ m, the detection limit (LOD) and quantitative limit (LOQ) are 6.34 µm and 21.1 µm, respectively. The sensor also shows excellent selectivity, the least interference from ordinary analytes, and at the same time, has storage stability, reproducibility, and repeatability of color. Phytic acid (PA), as a natural organic polyphosphoric acid compound, contains six phosphate ester bonds in its molecule, and has an extremely strong complexing ability. It can form stable complexes with various metal ions (such as Ce^3+^, Cu^2+^, etc.). Meanwhile, its unique structure can regulate the microstructure of materials, and increase their specific surface area and stability. It can also serve as a proton transfer medium to accelerate the progress of catalytic reactions. Lili Yang et al. [11] prepared a PA-PEDOT-CuNPs nanocomposite material modified glassy carbon electrode for enzyme-free glucose detection. PA-doped PEDOT provides a highly conductive and large specific surface area substrate, promoting the uniform growth of CuNPs and enabling direct oxidation of glucose. The detector has a linear range of 5–403 μM, a sensitivity of 79.27 μA μM^−1^ cm^−2^, a detection limit of 0.28 μM, and a response time of less than 4 s. Studies have shown that phytic acid can form stable complexes with Cu ions, regulate the morphology and properties of Cu-based materials, and be used to construct electrochemical sensors for detecting target molecules. Ce-based composites have shown great potential in peroxidase sensing due to their excellent catalytic performance.

Therefore, in this study, a multilayer nanostructure system based on GCE was adopted. Under a constant potential, Cu nanoparticles (with a particle size of 20–50 nm) were deposited on the glassy carbon electrode, providing a highly conductive substrate. The three-dimensional network structure can significantly increase the density of active sites. Due to the strong coordination between the phosphate group and Cu^2+^, the PA-Cu^2+^ coordination film can form a dense protective film, which not only prevents the oxidation and aggregation of Cu but also controls the electrostatic adsorption rate of Ce^3+^ on the film surface, making it an active center for the oxidation of H_2_O_2_. This study explored the synergistic catalytic mechanism among Ce^3+^, phytic acid and Cu^2+^@Cu by constructing an enzyme-free peroxidase sensor, and investigated the detection performance and practical application value of the sensor for H_2_O_2_, aiming to provide new ideas and experimental basis for the development of high-performance and low-cost peroxidase sensors.

## 2. Results

### 2.1. Results Analysis of H_2_O_2_ Electrochemical Sensor Based on Electroactive Ce^3+^-Phytic Acid-Cu^2+^@Cu Composite

Cu nanoparticles electrodeposited on glassy carbon electrode (GCE) were chosen as the signal source to obtain Cu/GCE modified electrode. Then, phytic acid (PA) and Ce^3+^ were alternately adsorbed onto the electrode via a self-assembly method to obtain Ce^3+^/PA-Cu^2+^@Cu/GCE. When H_2_O_2_ exists, Ce^3+^ is oxidized to achieve highly sensitive detection of H_2_O_2_ in milk. The specific test analysis is as follows.

#### 2.1.1. XRD Analysis of Ce^3+^-Phytic Acid-Cu^2+^@Cu Composite

XRD tests were conducted on Cu, PA-Cu^2+^@Cu, and Ce^3+^/PA-Cu^2+^@Cu nanocomposite powders, respectively, and the results are shown in Figure 1. The characteristic diffraction peaks of pure copper powder at 2θ = 337.3°, 42.5°, and 63.2° correspond to (111), (200), and (220) crystal planes of copper face-centered cubic structure (PDF #89-2838), respectively. The sharp peaks and high intensity indicate that the material has good crystallinity. When copper nanoparticles are contacted with PA to form PA-Cu^2+^@Cu composites, the intensity of the characteristic copper diffraction peak decreases obviously, which is attributed to the selective etching caused by the coordination of carboxylic acid groups in PA molecules with copper surfaces. By chelating Cu^2+^ to form soluble complexes, PA helps rebuild the surface of copper nanoparticles, thereby weakening their crystalline integrity.

After Ce^3+^ was introduced, the XRD pattern of Ce^3+^/PA-Cu^2+^@Cu showed that the diffraction peak intensity of Cu decreased further, which could be attributed to the synergistic effect of Ce^3+^ and PA. On the one hand, Ce^3+^ was adsorbed on PA-Cu^2+^@Cu surface by electrostatic interaction, forming the ternary Ce^3+^/PA-Cu^2+^@Cu complex. Ce^3+^/PA-Cu^2+^@Cu ternary complex enhances the chemical etching of copper; on the other hand, the hydrolysis of Ce^3+^ produces a local acidic microenvironment, which accelerates the oxidation dissolution of copper. Notably, no Ce-related diffraction peaks were observed in the diffraction pattern, suggesting that Ce^3+^ may exist as amorphous or highly dispersed nanoparticles, or embedded in PA-Cu^2+^ lattice structures. These results indicate that continuous Ce [12] treatment of PA can effectively adjust the surface structure of copper nanoparticles, and construct porous nanostructures through chemical etching and coordination, providing more active sites for subsequent electrocatalytic reactions.

#### 2.1.2. Infrared Spectrum Analysis of Ce^3+^-Phytic Acid-Cu^2+^@Cu Composite and Phytic Acid

Figure 2 shows infrared spectra of Cu, PA-Cu^2+^@Cu, Ce^3+^/PA-Cu^2+^@Cu nanocomposite powders and PA. Pure copper has no characteristic absorption peak in the range of 400–4000 cm^−1^, indicating that organic functional groups do not modify its surface. When Cu reacts with PA to form the PA-Cu^2+^@Cu composite material, the characteristic peak of PA appears in the spectrum. The symmetrical stretching vibration of the P=O bond in PA causes the peak at 1140 cm^−1^. After coordination with Cu, this peak is red-shifted to 1060 cm^−1^, resulting in an increase in the P=O bond length and a decrease in the vibrational frequency. In addition, the absorption peak at 988 cm^−1^ corresponds to the stretching vibration of P-O-C bond, reflecting the anchoring effect of PA molecule on the copper surface. After further introduction of Ce^3+^, the spectrum of Ce^3+^/PA-Cu^2+^@Cu shows a shift from 988 cm^−1^ to 1006 cm^−1^, which can be attributed to the coordination of Ce^3+^ with phosphate groups in PA. The high coordination number of Ce^3+^ forms polycomplexes with carboxylate and phosphate of PA through electrostatic interaction, resulting in the redistribution of electron cloud density and an increase in vibration frequency of P-O bond. Meanwhile, the hydroxyl peak intensity at 3420 cm [13] increases, indicating that the hydrolysis of Ce^−1^ introduces additional surface hydroxyl groups.

This result complements XRD analysis; IR spectra confirm the successful modification of PA and Ce^3+^, while XRD reveals surface structural changes in copper nanoparticles. They verify the controllable synthesis of Ce^3+^/PA-Cu^2+^@Cu electrode and provide a structural basis for optimizing electrocatalytic performance.

#### 2.1.3. Characterization of CV Cycle Curve of Ce^3+^-Phytic Acid-Cu^2+^@Cu Composite Modified Electrode

As shown in Figure 3, respectively, Cu/GCE, PA-Cu^2+^@Cu/GCE, Ce^3+^/PA-Cu^2+^@Cu/GCE for the working electrode, in the 5.0 mmol·L^−1^ K_3_[Fe(CN)_6_] of 0.1 mol·L^−1^ KCl in solution at 50 mV/s CV plot recorded at the scan rate of 0 to 1.0 V. Cu/GCE exhibits a pair of symmetrical redox peaks, indicating that the quasi-reversible reaction of [Fe(CN)_6_]^3−/4−^ couple occurs on Cu surface [14]. The peak oxidation current of PA-Cu^2+^@Cu/GCE decreases after PA is compounded. This is attributed to electrostatic repulsion between carboxylic acid groups in PA molecules, which hinders the diffusion-mediated mass transfer of [Fe(CN)_6_]^3−/4−^. It is noteworthy that the CV curve of Ce^3+^/PA-Cu^2+^@Cu/GCE shows a significantly improved electrical response, which is attributed to the fact that the high conductivity of Ce^3+^ accelerates the electron transport and reduces the charge transfer resistance (Rct)(verified by EIS); the complex formed by Ce^3+^ and PA provides more active sites for this pair of electrons and increases the effective area of the electrode.

XRD and FT-IR analysis confirmed this result: Ce^3+^ not only optimized the charge state at the electrode surface but also improved electron transfer efficiency through structural adjustment, laying the electrochemical foundation for subsequent H_2_O_2_ electrocatalytic detection.

#### 2.1.4. Characterization of Ce^3+^-Phytic Acid-Cu^2+^@Cu Composite Modified by EIS

Figure 4 shows electrochemical impedance spectroscopy (EIS) of Cu/GCE, PA-Cu^2+^@Cu/GCE, Ce^3+^/PA-Cu^2+^@Cu/GCE as working electrodes in 5.0 mmol·L^−1^ K_3_[Fe(CN)_6_] in 0.1 mol·L^−1^ KCl solution. The semicircular diameter in the high-frequency region corresponds to the charge-transfer resistance (Ret), while the diagonal in the low-frequency region reflects ion-diffusion behavior [15]. When Cu/GCE modified PA, the Ret of PA-Cu^2+^@Cu/GCE increased and the semicircular diameter increased by 76%. This phenomenon is due to the strong coordination of carboxylic acid groups in PA molecules: PA chelates with Cu^2+^ to form a dense, insulating layer that hinders the diffusion and mass transfer of [Fe(CN)_6_]^3−/4−^. In addition, electrostatic repulsion caused by the negative charge of PA further reduces mobility. It is worth noting that the Ret value for Ce^3+^/PA-Cu^2+^@Cu/GCE is 30% lower than that for pure Cu/GCE. This optimization is due to the synergistic effect of Ce^3+^ and PA. Ce^3+^ neutralizes the negative charge of PA by electrostatic adsorption and promotes the diffusion of charge pairs. Ce^3+^ complexation with PA promotes the surface reconstruction of copper and the formation of a porous structure, which increases the effective reaction area. These results are consistent with the CV analysis, which verifies that Ce^3+^ can significantly improve the electrode’s electron transfer efficiency through charge modulation, conductivity enhancement, and structure optimization.

### 2.2. Feasibility of Ce^3+^-Phytic Acid-Cu^2+^@Cu Composite Electrochemical Sensor for H_2_O_2_ Detection

#### 2.2.1. Feasibility Analysis of Ce^3+^-Phytic Acid-Cu^2+^@Cu Composite Electrochemical Sensor for H_2_O_2_ Detection Verified by Cyclic Voltammetry

As shown in Figure 5A, the electrochemical responses of bare GCE, Cu/GCE, PA-Cu^2+^@Cu/GCE, and Ce^3+^/PA-Cu^2+^@Cu/GCE modified electrodes in H_2_O_2_ solution were tested by CV in 0.1 mol L^−1^ PBS containing 50 μmol H_2_O_2_. The test results show that the oxidation peak (E_Pa_) of bare GCE is approximately 0.45 V, and the reduction peak (E_Pc_) is about −0.05 V. The peak potential difference ΔE_P_ is approximately 0.50 V. No characteristic redox peaks were observed within the scanning range, indicating that it lacks electrocatalytic activity for the target analyte. The Cu/GCE electrode shows a distinct oxidation peak, with the peak current increasing. E_Pa_ is approximately 0.40 V, E_Pc_ is about 0.00 V, and ΔE_P_ ≈ 0.40 V. This is attributed to the excellent electrocatalytic performance of Cu nanoparticles. However, it can be more obviously observed that after the PA-Cu^2+^@Cu/GCE nanoparticles combined with phytic acid, E_Pa_ positively shifted to 0.50 V, E_Pc_ negatively shifted to −0.10 V, ΔE_P_ ≈ 0.60 V, and at the same time, the CV response value to H_2_O_2_ showed a downward trend. This might be due to the fact that PA forms a non-conductive molecular film on the surface of Cu. It is worth noting that H_2_O_2_ exhibits the maximum redox peak at Ce^3+^/PA-Cu^2+^@Cu/GCE, with the peak current being the highest among all electrodes. The oxidation peak current approaches 35 μA, and the reduction peak current also exceeds −10 μA. The strongest indicates that the introduction of Ce^3+^ further enhances the catalytic activity and electron transfer rate. The results above confirm that Ce^3+^/PA-Cu^2+^@ Cu/GCE has the best catalytic effect for the determination of H_2_O_2_.

The histogram in Figure 5B shows the trend of peak oxidation current (IPa) for each electrode: Ce^3+^/PA-Cu^2+^@Cu/GCE has IPa values 12 times higher than bare GCE, 2 times higher than Cu/GCE, and 3.4 times higher than PA-Cu^2+^@Cu/GCE, which means that Cu nanomaterials modified on the surface of glassy carbon electrode not only improve the electron conduction efficiency, but also provide a rich attachment site for PA, which can be used to coordinate with Cu/GCE superficial Cu binding, and while further chelating Cu^2+^, Ce^3+^ has excellent redox activity and catalytic performance. It is enriched on the electrode surface by combining with the remaining phosphoric acid group of PA, and this not only increases the number of catalytic sites, but also uses Ce^3+^ as the high catalytic activity of itself further accelerates the redox reaction of target molecules and finally makes the peak current reach the maximum value. The graph shows the current trend in variation during electrode preparation and H_2_O_2_ detection, demonstrating that the experiment is feasible.

#### 2.2.2. Feasibility Analysis of Ce^3+^-Phytic Acid-Cu^2+^@Cu Composite Electrochemical Sensor for H_2_O_2_ Detection Verified by Square Wave Voltammetry

Figure 6 shows SWV images of GCE, Cu/GCE, PA-Cu^2+^@Cu/GCE, Ce^3+^/PA-Cu^2+^@Cu/GCE in H_2_O_2_ solution to Ag/AgCl saturation KCl as a reference electrode. It can be observed that when only GCE bare electrode is used, the corresponding SWV curve does not have oxidation peak current, which indicates that bare electrode has no electrochemical activity; for the Cu/GCE electrode, an oxidation peak was shown at 0.20 V in SWV, indicating that Cu physically adheres to GCE, which has a clear signal for the detection of H_2_O_2_; when PA and Cu/GCE form PA-Cu^2+^@Cu/GCE electrode, the oxidation peak site is at 0.23 V, and the oxidation peak current value in SWV curve decreases, which indicates that the conductivity of Cu^2+^ decreases due to PA covering, which is consistent with the trend expressed in CV image. Finally, we can clearly observe that the oxidation peak on the Ce^3+^/PA-Cu^2+^@Cu/GCE electrode at the 0.26 V oxidation peak site is the largest, which indicates that the appearance of Ce^3+^ produces electron transfer between ion valence states. Meanwhile, the introduction of Ce^3+^/PA-Cu^2+^@Cu/GCE enhances the conductivity of Cu^2+^ nanomaterials, which is also consistent with CV images, indicating that the catalytic effect of H_2_O_2_ determination on Ce^3+^/PA-Cu^2+^@Cu/GCE electrode is the best [16].

### 2.3. Optimization of Preparation Conditions for Ce^3+^-Phytic Acid-Cu^2+^@Cu Composite Electrochemical Sensor

#### 2.3.1. Time Optimization Conditions for Electrodeposition of Cu

According to Section 4.6.1, the time for Cu electrodeposition is optimized. Figure 7 shows the SWV diagram of time optimization for the electrodeposition of Cu and the broken-line diagram of oxidation peak current (Ip) versus time (t). The figure shows that the oxidation peak current of Cu/GCE gradually increases with increasing deposition time from 100 s to 700 s. This phenomenon is attributed to the nucleation and growth of copper nanoparticles during electrodeposition. In the initial stage, copper nucleates on the GCE surface in an island-like structure, and the density of active sites is relatively low. With the extension of deposition time, copper particles gradually agglomerate to form continuous thin films, which makes the effective reaction area increase. After deposition time exceeds 700 s, the oxidation peak current begins to decrease. This transition is due to structural defects caused by excessive thickening of the deposited layer, increase in porosity and coarsening of grains in the thick film, thus reducing the number of active sites; increase in porosity between the deposited layer and GCE will also reduce the number of active sites; and weakening of bonding force between the deposited layer and GCE will lead to partial copper detachment. Therefore, 700 s was determined to be the optimal electrodeposition time, at which the electrode exhibited the highest catalytic oxidation efficiency for H_2_O_2_.

#### 2.3.2. Optimization of Soaking Time of Phytic Acid Solution

According to Section 4.6.2, the immersion time of the phytic acid solution was optimized. The results are shown in Figure 8. With the reaction time increasing from 10 min to 60 min, the peak oxidation current of PA-Cu^2+^@Cu/GCE gradually decreased, reflecting the layer-by-layer adsorption of PA molecules on the copper surface. When the reaction time exceeds 45 min, PA molecules form a multilayer adsorption via chelation, forming a dense, insulating layer on the copper surface that hinders the diffusion and mass transfer of H_2_O_2_. When the reaction time reaches 60 min, the oxidation peak current tends to stabilize, indicating that the coordination etching process of PA and copper reaches a dynamic equilibrium. At this time, PA molecules thoroughly coat Cu nanoparticles through coordination, forming PA-Cu^2+^@Cu structure. When the reaction time reaches 80 min, the current shows no significant change, indicating that the PA etching process has been completed and that excessive PA adsorption has no further effect on the electrode characteristics. Therefore, 60 min is selected as the optimal reaction time for PA-Cu^2+^@Cu/GCE.

#### 2.3.3. Ce^3+^ Recombination Time Optimization

According to Section 4.6.3, the reaction time of Ce^3+^ is optimized. The results are shown in Figure 9. When the modification time is 10–60 min, the peak oxidation current of Ce^3+^/PA-Cu^2+^@Cu/GCE gradually increases, reflecting the layer-by-layer adsorption process on its surface. Ce^3+^ is adsorbed on the carboxylic acid group of PA through electrostatic action at 10–30 min, forming monolayer coverage. When the modification time exceeds 45 min, Ce^3+^ forms a ternary complex with PA through coordination, and the electron mobility increases. When the modification time reached 60 min, the oxidation current peak reached its maximum, indicating that Ce^3+^ adsorption had saturated. When the modification time was extended to 80 min, the current decreased slightly, due to decreased active sites resulting from excessive Ce^3+^ aggregation, and the rate was affected. Therefore, Ce^3+^ reaction time of 60 min was selected as the optimum reaction time.

#### 2.3.4. Optimization of Reaction Time of Ce^3+^-Phytic Acid-Cu^2+^@Cu Composite to H_2_O_2_

According to Section 4.6.4, select a different time to optimize H_2_O_2_, carry out SWV test on it and plot the curve. The results are shown in Figure 10. In 5–10 min, the oxidation of Ce^3+^ increases the charge density at the electrode surface, which is beneficial for the diffusion and mass transfer of H_2_O_2_. When the reaction time was 15 min, the oxidation current peak reached its maximum, indicating that the reaction between Ce^3+^ and H_2_O_2_ had reached dynamic equilibrium. At this time, Ce^3+/4+^ mixed oxide layer was formed on the electrode surface, which accelerated the electron transfer during the redox cycle. When the reaction time was 30 min, the current decreased slightly due to surface passivation and the aggregation and reduction of active sites caused by excessive Ce^4+^ accumulation. Therefore, 15 min was chosen as the reaction time of H_2_O_2_.

### 2.4. Linear Range and Detection Limit Analysis of Ce^3+^/PA-Cu^2+^@Cu/GCE for H_2_O_2_ Detection

To systematically evaluate the performance of Ce^3+^/PA-Cu^2+^@Cu/GCE sensors for detecting H_2_O_2_, electrodes were tested over the H_2_O_2_ concentration range of 0.1 μmol·L^−1^ to 100 μmol L^−1^ using optimized experimental parameters. The study was conducted using the DPV method in 0.1 mol·L^−1^ PBS (pH 6.8) and the results are shown in Figure 11. It should be noted that the peak oxidation current (vs. Ag/AgCl) of Ce^3+^/PA-Cu^2+^@Cu/GCE electrode at 0.21 V increased significantly with the increase in H_2_O_2_ concentration. In the range of 0.1 μmol·L^−1–^100 μmol·L^−1^, the peak oxidation current (IPa) has a good linear relationship with H_2_O_2_ concentration, and its linear equation is as follows:

$$IPa(\mu A)=1.46C({\mu mol\cdot L}^{-1})+13.34$$

Correlation coefficient R^2^ = 0.99. At S/N = 3, the limit of detection (LOD) is 0.89 μmol∙L^−1^, which is much lower than traditional enzyme-based sensors and has a wider detection range than many nanomaterial-based sensors. The detection limit reaches micromolar value, which is attributed to the high conductivity of copper nanoparticles and the synergistic effect of Ce^3+^/PA coordination network: copper nanoparticles provide a direct electron transfer pathway, Ce^3+^/Ce^4+^ redox coupling, H_2_O_2_ adsorption and activation enhance the electron transfer pathway. In addition, the sensor has a high sensitivity of 1.46 μA/(μmol∙L^−1^), which is attributed to the direct modification of copper nanoparticles as a signal source on the electrode surface, providing a direct electron transport path, so that electrons can be transferred more efficiently, thus enhancing the signal output, making the sensitivity better, indicating that the surface adsorption step controls the catalytic process.

### 2.5. Interference Substance Pair H_2_O_2_ Impact of Test Results

According to Section 4.7, selectivity tests were conducted on the sensor to examine whether the Ce^3+^/PA-Cu^2+^@Cu/GCE detection of H_2_O_2_ is affected by other substances. The results are shown in Figure 12, and the current response of the oxidation of interfering ions is lower than that of 10 μA, and H_2_O_2_ can be achieved at 56 μA. Obviously, with Ce^3+^/PA-Cu^2+^ @Cu/GCE constructed using the prepared composite nanomaterials, the sensor can effectively resist the interference of different substances, but also can selectively detect H_2_O_2_. Ce^3+^/PA-Cu^2+^@Cu/GCE energy and H_2_O_2_ for the specific reaction, and the change of the electrochemical signal of the sensor shows that the electrochemical sensor established in this experiment has a relatively good selectivity.

### 2.6. Reproducibility and Stability Analysis of Ce^3+^/PA-Cu^2+^@Cu/GCE

The reproducibility and stability of Ce^3+^/PA-Cu^2+^@Cu/GCE electrode were tested. See 4.9 for details. The results are shown in Figure 13. According to image (A), the current response differences among sensors are minimal, with an RSD of only 1.013%, indicating excellent reproducibility. According to the image (B), the peak current signal of H_2_O_2_ gradually decreases with the increase in storage time, and the decrease in amplitude is the largest in the first cycle. Then, the reduction in speed slows down, but even on the 28th day, the H_2_O_2_ current signal is still about 84%. The current retention rate of the sensor is 89.0% after 7 days in 60 °C oven and 88.6% after 7 days in 90% humidity. This study shows that Ce^3+^/PA-Cu^2+^@Cu/GCE has relatively good reproducibility and stability.

## 3. Discussion

In this study, a constant potential deposition method was used GCE surface preparation of copper nanoparticles, complexing Cu^2+^ by phytic acid (PA) etching was formed into a coordination film, and then Ce^3+^ was immobilized to construct a three-layer modified electrode. Through XRD, FT-IR characterization confirms the successful synthesis of the material by CV, EIS the electron transfer characteristics of the electrode interface were analyzed. Optimize and determine the best process conditions: cu time of electrodeposition 700 s, PA embellishment 60 min, Ce^3+^ coordination 60 min, H_2_O_2_ Reaction 15 min. The detection linearity range of the sensor for hydrogen peroxide is 0.1–100 μmol/L, detection limit is 0.89 μmol/L, good selectivity, sensitivity and reproducibility (RSD = 1.013%) and stability (retention of 84% response at 28 days).

## 4. Materials and Methods

### 4.1. Materials and Instruments

Sodium hydroxide (NaOH), ammonium sulfate ((NH_4_)_2_SO_4_), H_2_O_2_ (36–38%), phytic acid (PA), cerium nitrate hexahydrate (Ce(NO_3_)_3_), phosphate buffer solution (PBS, pH = 6.8).

X-ray diffractometer (Ultima IV, Rigaku Corporation, Tokyo, Japan), Fourier Transform Infrared spectrometer (NICOLETIS10, Thermo Fisher Scientific, Waltham, MA, USA), and electrochemical workstation (CHI 660E, CH Instruments Inc., Shanghai, China).

### 4.2. Pretreatment of Working Electrode

Polishing bare glassy carbon electrode (GCE): take a little polishing powder (Al_2_O_3_, particle size 0.05 μm) on the polishing plate (suede), drop a small amount of deionized water, and mix well with the insulating part of the glassy carbon electrode. After Al_2_O_3_ suspension is formed on a polishing disc, holding the glassy carbon electrode vertically, exerting uniform force on the elbow of the arm to make the glassy carbon electrode move slowly in suede, wherein the path is an ‘8’ shape, polishing the glassy carbon electrode for 2–3 min, grinding clockwise for about 100 circles, performing ultrasonic treatment in deionized water and ethanol solution for 30 s, respectively (power is 500 w), drying the electrode at room temperature after removing Al_2_O_3_ powder on the surface of the glassy carbon electrode and finishing pretreatment. Go to the next step [17].

### 4.3. Construction of Electroactive Ce^3+^-Phytic Acid-Cu^2+^@Cu Composite Electrochemical Sensor

#### 4.3.1. Construction of Cu/GCE

Pretreat the electrode according to the Section 4.2. Immerse the pretreated polished GCE electrode in a mixture of 0.01 mol·L^−1^ CuSO_4_ and 0.5 mol·L^−1^ (NH_4_)_2_SO_4_, then apply a constant potential of−0.25 V (vs. Ag/AgCl) using potentiostatic chronoamperometry (It), and deposit for 700 s to form a copper nanoparticle-modified layer.

#### 4.3.2. Construction of Phytic Acid-Cu^2+^@Cu/GCE Composite

The resulting Cu/GCE electrode was immersed in 10 mM phytic acid (PA) solution and incubated in a constant-temperature incubator at 37 °C for 1 h. After the reaction is complete, the resulting composite is gently washed with ultrapure water to remove unbound free phytic acid molecules and to prepare PA-Cu^2+^@Cu/GCE composites.

#### 4.3.3. Construction of Ce^3+^/Phytic Acid-Cu^2+^@Cu/GCE Composite Sensor

The PA-Cu^2+^@Cu/GCE electrode was immersed in 20 mM Ce(NO_3_)_3_ solution, incubated at 37 °C for 1 h, and washed with ultrapure water to remove excess ions adsorbed on the surface, yielding the Ce^3+^/PA-Cu^2+^@Cu/GCE ternary composite electrochemical sensor.

### 4.4. Testing and Characterization of Cerium-Based Nanocomposites

#### 4.4.1. X-Ray Diffractometer (XRD) Determination Method

To analyze the surface morphology of the cerium-based nanocomposites prepared above, X-ray diffraction (XRD) was used. XRD test can effectively characterize the crystal structure, elemental composition, and micro-morphology of adsorption materials, and qualitatively and quantitatively analyze the materials [18]. Phase analysis was performed using an Ultima IV X-ray diffractometer from Rigaku, equipped with a Cu Kα source (λ = 1.5406 A). The parameters were set as follows: the scanning range 2θ was 5°~80°, the step size was 0.02°, and the scanning speed was 4°/min. Experimental data were analyzed using JADE 6.5 software and integrated with the International Crystal Database (ICSD) for matching and structural refinement.

#### 4.4.2. Fourier Transform Infrared (FT-IR) Determination Method

Fourier Transform Infrared spectroscopy (FT-IR) can qualitatively identify chemical components, reveal molecular binding characteristics, and functional group structure information [19]. In this study, the cerium-based nanocomposites were tested by a Thermo Scientific NICOLETIS10 Fourier Transform Infrared spectrometer equipped with DTGS detector and KBr beam splitter, scanning range 4000–200 cm^−1^, resolution 4 cm^−1^, scanning cycle 32 times. Cerium-based nanocomposites were tested. The sample was prepared by the KBr tabletting method: 1–2 mg of adsorbent powder and 200 mg of dried KBr crystals were placed in an agate mortar, ground thoroughly, and stirred (10 min) to a particle size of 2 μm, and then maintained at a pressure of 10 MPa for 3 min using a tabletting machine until transparent flakes were formed. The prepared samples were pretreated in a vacuum oven (60 °C, 2 h) for spectrum acquisition. At the end of the experiment, Origin 2021 software was used to perform baseline correction and peak position resolution on the data, and functional group assignment analysis was performed in combination with the OMNIC spectrum library. To eliminate external interference, all studies were conducted in a test chamber at constant temperature and humidity (25 ± 1 °C, 45% RH), and the average spectrum of each sample was calculated from the three spectral bands.

#### 4.4.3. Determination Method of Electrochemical Cyclic Voltammetry (CV)

A three-electrode system was used, in which a glassy carbon electrode (GCE, ϕ = 3 mm) modified by cerium-based nanocomposite was used as the working electrode, Ag/AgCl as reference electrode, platinum column as auxiliary electrode, and 0.1 mol·L^−1^ KCl solution (containing 5.0 mmol·L^−1^ K_3_[Fe(CN)_6_]) as electrolyte. Cyclic voltammetry (CV) was performed using a CHI 660E electrochemical workstation with a scan rate of 100 mV·s^−1^ and a measurement potential range of −0.2 V to 1 V (vs. Ag/AgCl). Before testing, each electrode was run through an unloaded electrolyte to a stable baseline, and an average curve was recorded over three consecutive cycles for formal testing.

#### 4.4.4. Electrochemical AC Impedance (EIS) Measurement Method

A three-electrode working system (same as Section 4.4.3) was adopted, electrochemical impedance spectroscopy (EIS) was used to characterize the electrode interface resistance, and 5.0 mol L^−1^ K_3_[Fe(CN)_6_] and 5.0 mol L^−1^ K_4_[Fe(CN)_6_] 0.1 mol L^−1^ KCl were used as electrolyte solution, frequency range was selected from 10^−2^ to 10^5^ Hz, AC amplitude was 5 mV, and open circuit potential was 0.2 V (vs. Ag/AgCl). RLC equivalent circuit model, including solution resistance (Rs), charge transfer resistance (Rct), and double layer capacitance (Cdl) was fitted, and impedance spectrum was analyzed by ZSimpWin software. By comparing changes in the charge transfer resistance (Rct) at the electrode surface with the image characteristics, the influence of the modified layer on the electron transfer process at the electrode interface was evaluated, and the loading state of the functional materials was preliminarily determined. Calibrate the electrochemical workstation before testing, and perform cyclic voltammetry scanning (100 mV·s^−1^) in blank electrolyte to confirm that the background current of electrodes is stable (≤10 μA). Test each electrode in parallel three times and take the average value.

### 4.5. Feasibility of Cerium-Based Nanocomposite Electrochemical Sensor for H_2_O_2_ Detection

#### 4.5.1. Cyclic Voltammetry to Verify the Feasibility of Cerium-Based Nanocomposite Electrochemical Sensor for H_2_O_2_ Detection

In preliminary experiments, a pH 6.8 and CV scan rate of 100 mV·s^−1^ were determined for phosphate-buffered saline (PBS). CV was used to evaluate the catalytic performance of different modified electrodes for H_2_O_2_, and the test was performed in a three-electrode system (as in Section 4.4.3). The electrolyte was 50 μmol L^−1^ H_2_O_2_ in 0.1 mol L^−1^ PBS (pH 6.8), cycled 5 times over the potential range of −0.2 V to 0.6 V (vs. Ag/AgCl) (third curve for analysis). To verify the feasibility of detection, different modified electrodes were compared simultaneously. The data were recorded using CHI 660E workstation software, and the experimental results were plotted using Origin 2021 software.

#### 4.5.2. Square Wave Voltammetry to Verify the Feasibility of Cerium-Based Nanocomposite Electrochemical Sensor for H_2_O_2_ Detection

Square wave voltammetry (SWV) was used to evaluate the detection performance of the modified electrode for H_2_O_2_. The test was carried out in a three-electrode system (same as Section 4.4.3), and the electrolyte solution was 0.1 mol L^−1^ PBS (pH = 6.8) containing 50 μmol L^−1^ H_2_O_2_. SWV parameters were set as follows: potential window: −0.3 V~0.3 V (vs. Ag/AgCl), pulse amplitude: 4 mV, sweep amplitude: 25 mV, frequency: 15 Hz. To verify the feasibility of detection, a comparison experiment using different modified electrodes with the same index was conducted. The oxidation peak currents were recorded on a CHI 660E electrochemical workstation, baseline-corrected, and peak-fitted using Origin 2021.

### 4.6. Optimization of Construction Conditions of Electroactive Ce^3+^-Phytic Acid-Cu^2+^@Cu Composite Electrochemical Sensor

To obtain the best analytical performance of the H_2_O_2_ biosensor method based on the Ce^3+^/PA-Cu^2+^@Cu/GCE electrode, under the pre-experimental conditions, the substrate solution was selected as a PBS buffer solution (pH 6.8) with a H_2_O_2_ concentration of 50 μmol∙L^−1^, and the SWV parameters were set to potential window: −0.3 V~0.3 V (vs. Ag/AgCl), pulse amplitude: 4 mV, scan amplitude: 25 mV, and frequency: 15 Hz. Under the conditions, different factors were optimized, respectively.

#### 4.6.1. Time Optimization of Cu Electrodeposition

Cu/GCE electrode was prepared according to Section 4.3.1. The oxidation peak current value of SWV in the substrate solution was used as index. The immersion time of fixed phytic acid solution was 60 min, Ce^3+^ complex time was 60 min, H_2_O_2_ reaction time was 10 min, and different electrodeposition times (100 s, 200 s, 300 s, 400 s, 500 s, 600 s, 700 s, 800 s) were optimized.

#### 4.6.2. Optimization of Soaking Time of Phytic Acid Solution

PA-Cu^2+^@Cu/GCE electrode was prepared by Section 4.3.2. Taking the oxidation peak current value of SWV to substrate solution as index, the fixed electrodeposition time of Cu was 600 s, Ce^3+^ recombination time was 60 min, and H_2_O_2_ reaction time was 10 min. Different immersion times (10 min, 20 min, 300 min, 400 min, 50 min, 60 min, 70 min, 80 min) of phytic acid solution were optimized, respectively.

#### 4.6.3. Ce^3+^ Recombination Time Optimization

Ce^3+^/PA-Cu^2+^@Cu/GCE electrode was prepared by Section 4.3.3. Taking the oxidation peak current value of SWV to substrate solution as index, the fixed electrodeposition time of Cu was 600 s, the immersion time of phytic acid solution was 60 min, and the reaction time of H_2_O_2_ was 10 min. The Ce^3+^ recombination times (10 min, 20 min, 300 min, 400 min, 50 min, 60 min, 70 min, 80 min) were optimized.

#### 4.6.4. Optimization of Reaction Time of Ce^3+^-Phytic Acid-Cu^2+^@Cu Composite to H_2_O_2_

On the basis of the pre-experiment, the oxidation peak current of SWV to the substrate solution was taken as index, the time of Cu electrodeposition was fixed at 600 s, the immersion time in phytic acid solution was 60 min, and the Ce^3+^ recombination time was 60 min. The reaction time with H_2_O_2_ substrate was optimized, respectively (5 min, 10 min, 15 min, 20 min, 25 min, 30 min).

### 4.7. The Linear Range and Detection Limit of Cerium-Based Nanocomposite Electrochemical Sensors for H_2_O_2_ Detection

To explore the analytical performance of the sensor for H_2_O_2_ detection, under the optimized optimal experimental conditions, the cerium-based nanocomposite electrochemical sensor was placed in a 10 mmol∙L^−1^ PBS buffer (pH = 6.8) containing different concentrations of H_2_O_2_ (ranging from 0.1 μmol∙L^−1^ to 100 μmol∙L^−1^) solution for reaction. The electrochemical signal was detected by differential pulse voltammetry (DPV), and the oxidation peak current value was recorded. Each concentration point was tested in parallel three times, and the average response value was taken. DPV test parameters: potential window: −0.2 V to 0.8 V (vs. Ag/AgCl); pulse amplitude: 50 mV; scanning step size: 4 mV; pulse width: 50 ms.

### 4.8. The Influence of Interfering Substances on the Detection Results of H_2_O_2_

In practical applications, selectivity is one of the key performance indicators of sensors. To evaluate the specificity of the sensor in detecting H_2_O_2_ in this study and determine whether other substances would interfere with it, we selected representative interfering substances related to common components in milk beverages, including common inorganic ions (Ca^2+^, Mg^2+^), glucose, uric acid, lipids, proteins and ascorbic acid. Prepare 10 mmol∙L^−1^ PBS buffer solution containing H_2_O_2_ (pH = 6.8) and buffer solution containing interfering ions, respectively. Make the concentration ratio of the interfering substance to H_2_O_2_ 100:1. In the study, differential pulse voltammetry (DPV) was used, and the parameters were the same as those in Section 4.7. Before each test, the sensor is stabilized in the solution for 30 s, and then a DPV scan is performed and the oxidation peak current value is recorded. Analyze the results to determine the influence of different interfering substances on the sensor.

### 4.9. Detection of Reproducibility and Stability of Cerium-Based Nanocomposite Electrochemical Sensors

To evaluate the consistency among sensor packages, six glassy carbon electrodes (GCE, ϕ = 3 mm) were selected for this experiment and pretreated according to the method described in 4.2. Cerium-based nanocomposite electrochemical sensors were fabricated in parallel according to the optimized parameters, and DPV analysis was conducted in PBS electrolyte containing 20 μmol∙L^−1^ H_2_O_2_ (pH value 6.8) (with a potential difference window of 0.2–0.8 V (vs. Ag/AgCl), pulse amplitude 50 mV, scanning step size 4 mV. Calculate the relative standard deviation (RSD) of the response currents of the six electrodes to check the reproducibility.

In addition, the improved electrode was sealed in silica gel desiccant and stored in a light-proof area (25 ± 2 °C, humidity ≤ 40% RH), activated with blank PBS solution, and the current response to PBS buffer solution containing 20 μmol∙L^−1^ H_2_O_2_ solution was measured on days 1, 7, 14, 21, and 28. Use the same DPV parameters. Long-term stability is evaluated by calculating the current retention rate.

## Figures and Tables

**Figure 1:**
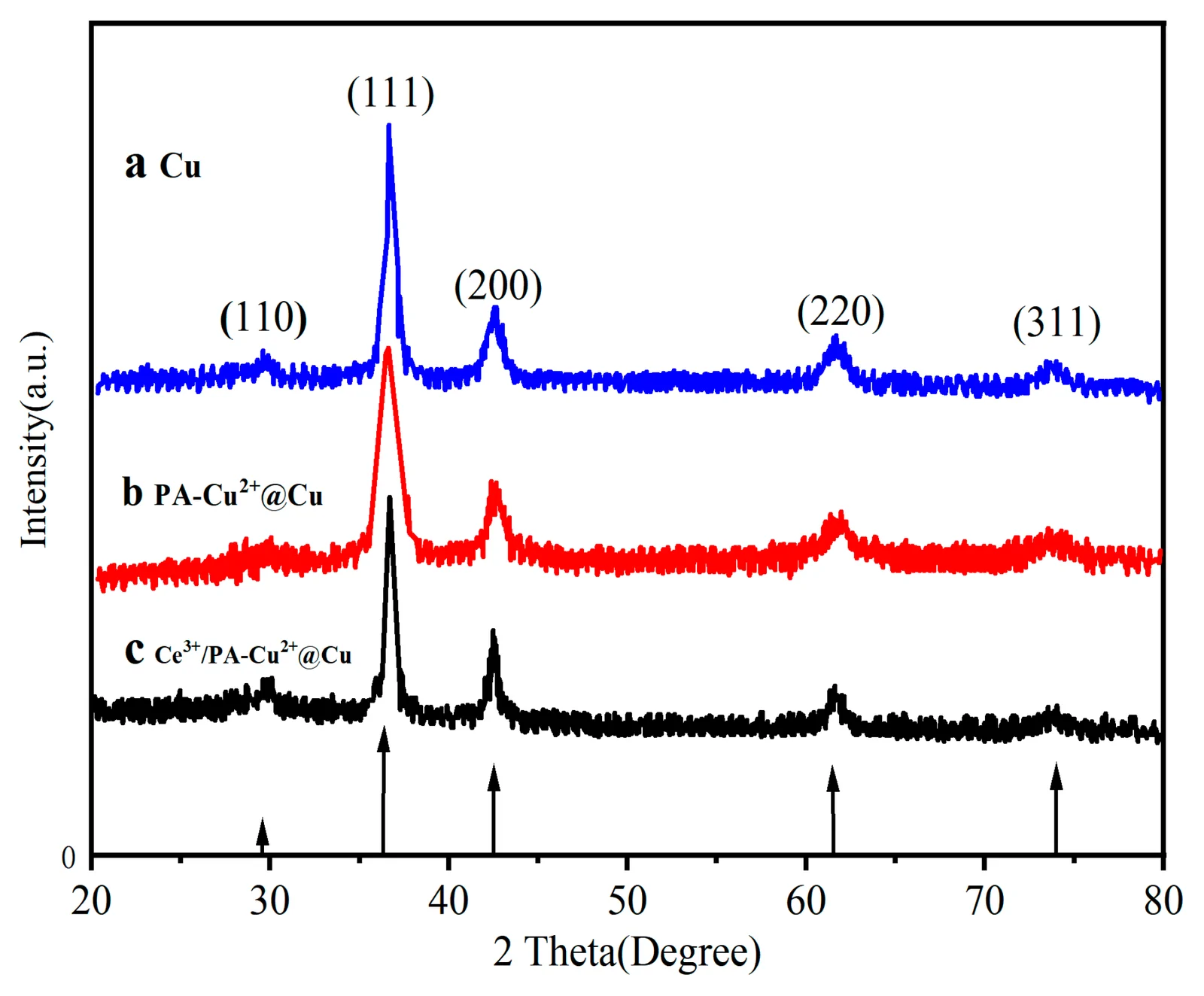
XRD spectra of Cu, PA-Cu^2+^@Cu, Ce^3+^/PA-Cu^2+^@Cu.

**Figure 2:**
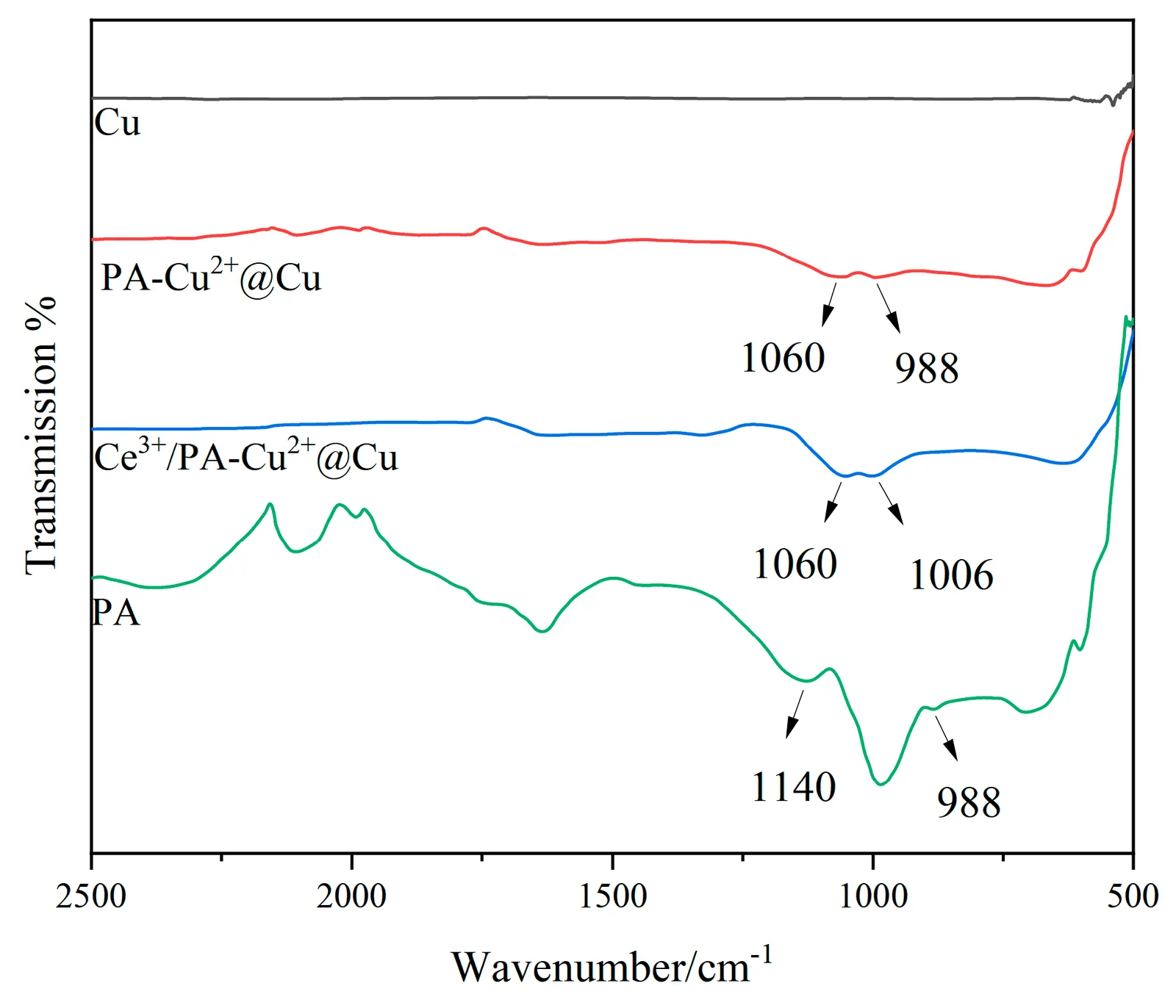
FTIR spectra of Cu, PA-Cu^2+^@Cu, Ce^3+^/PA-Cu^2+^@Cu and PA.

**Figure 3:**
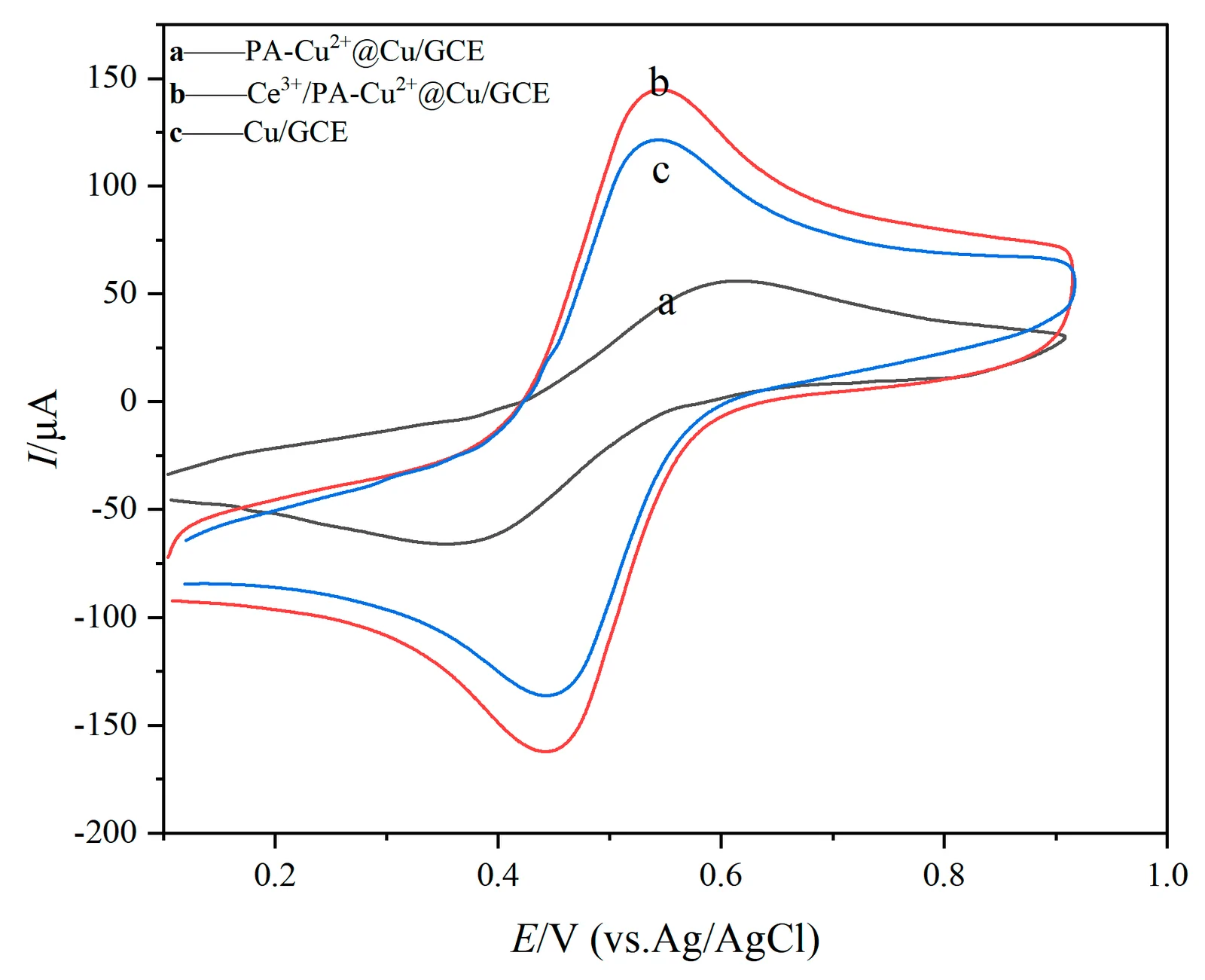
CV cycling profiles of Cu/GCE, PA-Cu^2+^@Cu/GCE, Ce^3+^/PA-Cu^2+^@Cu/GCE.

**Figure 4:**
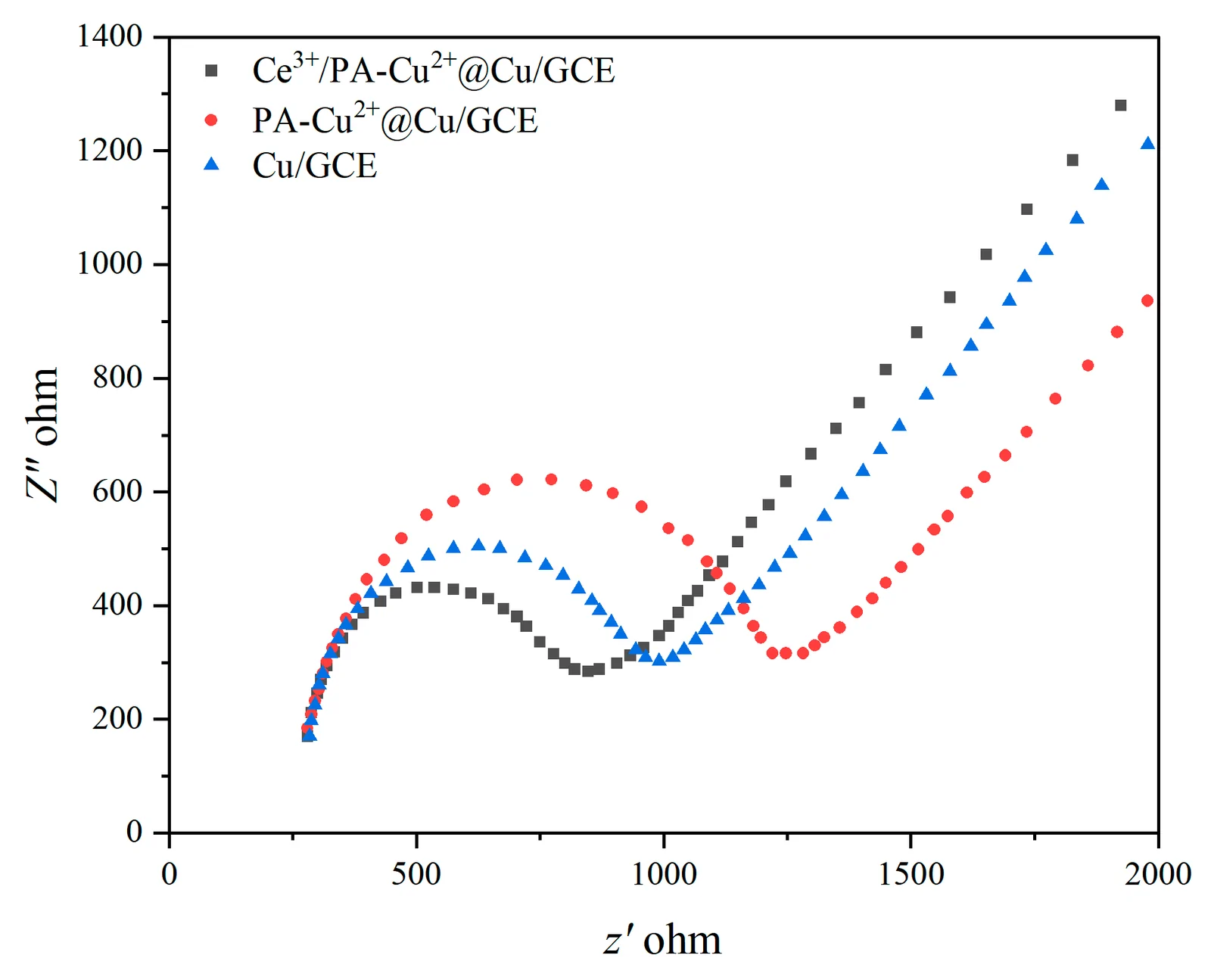
EIS curves for Cu/GCE, PA-Cu^2+^@Cu/GCE, Ce^3+^/PA-Cu^2+^@Cu/GCE.

**Figure 5:**
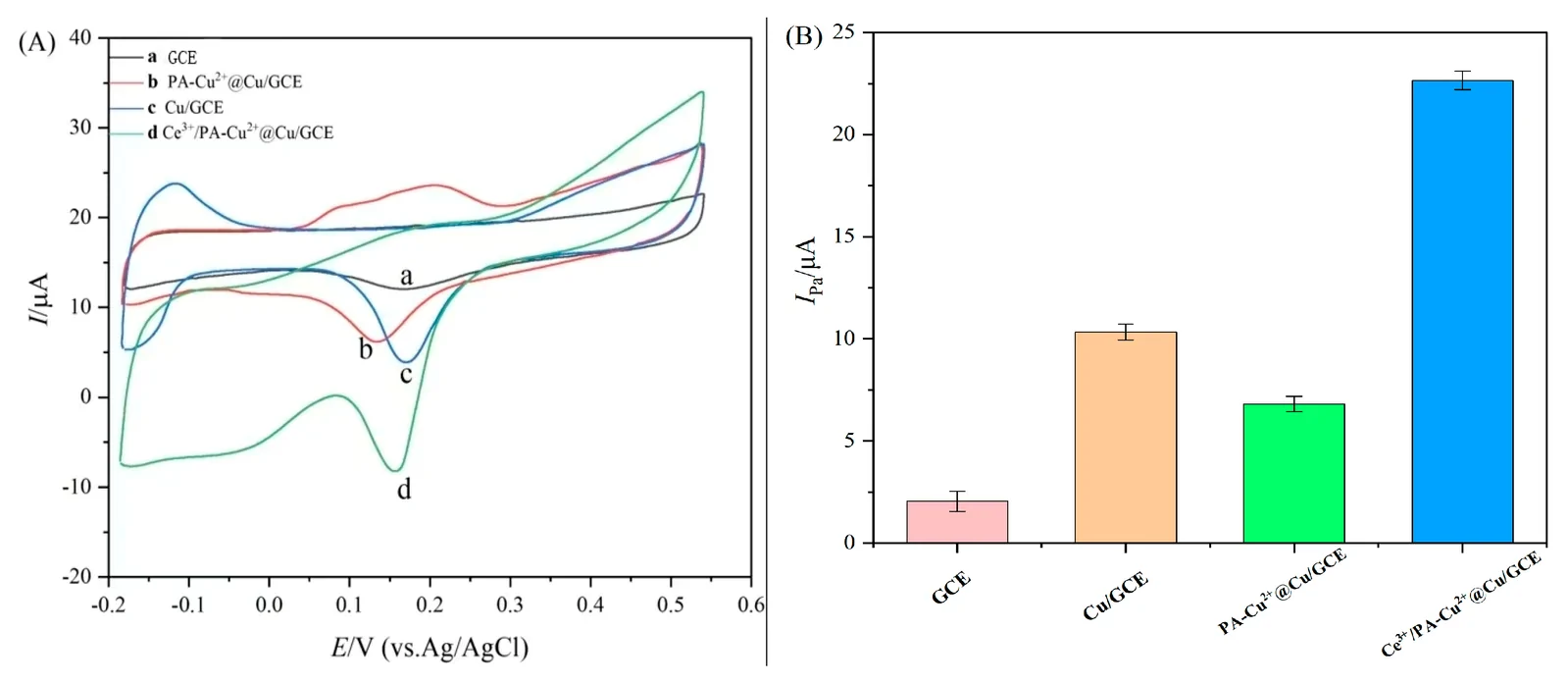
(**A**) CV plots of GCE, Cu/GCE, PA-Cu^2+^@Cu/GCE, Ce^3+^/PA-Cu^2+^@Cu/GCE in H_2_O_2_ solution and (**B**) histogram of oxidation peak current value (IPa).

**Figure 6:**
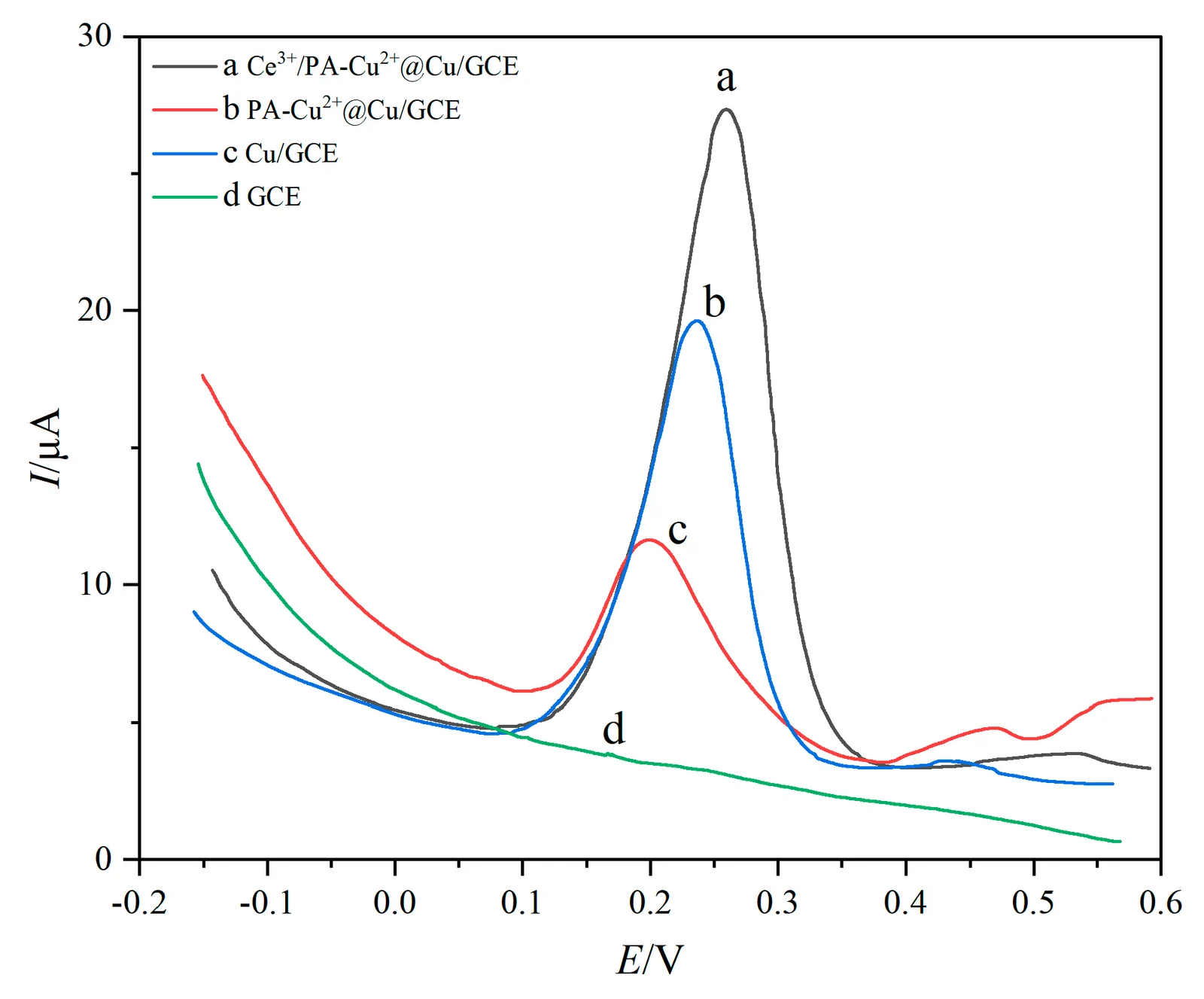
SWV profiles of GCE, Cu/GCE, PA-Cu^2+^@Cu/GCE, Ce^3+^/PA-Cu^2+^@Cu/GCE in H_2_O_2_ solution.

**Figure 7:**
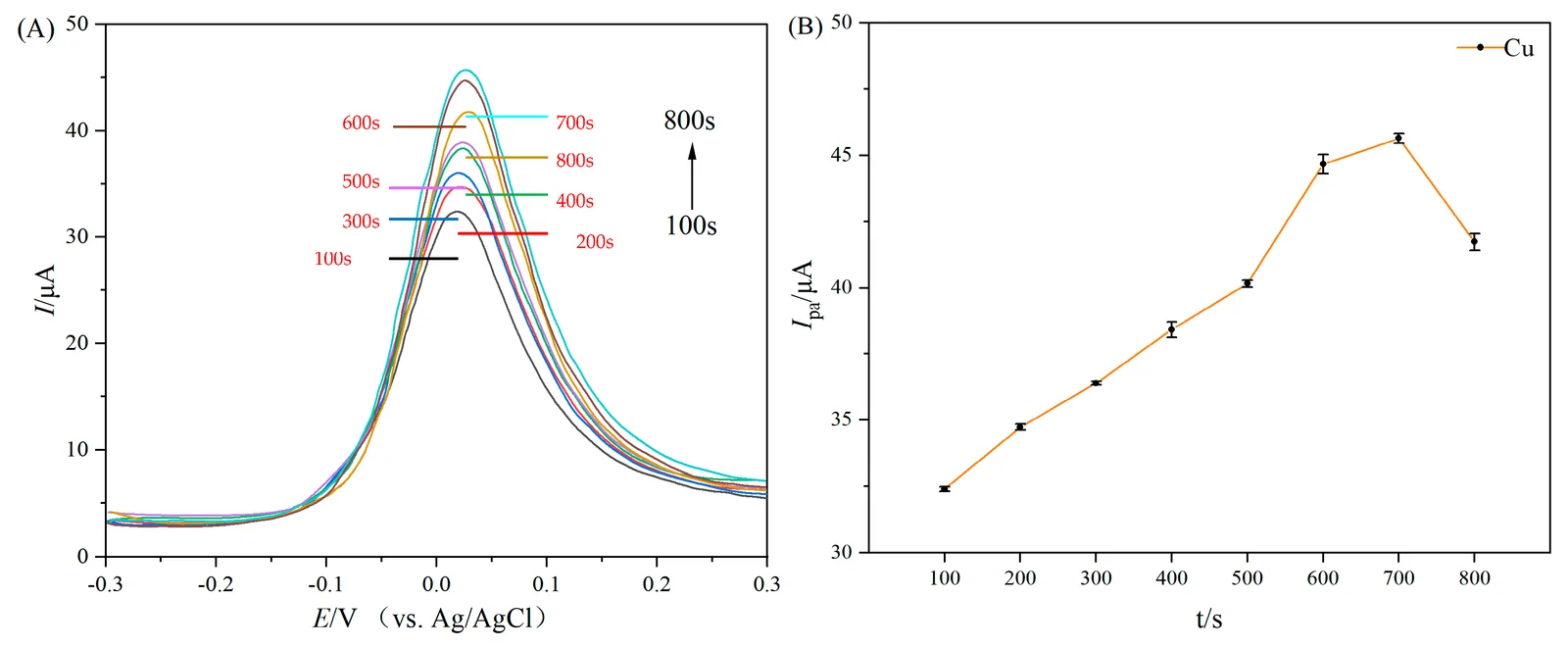
(**A**) Time-optimized SWV plot of electrodeposited Cu and (**B**) its oxidation peak current value (Ip) plotted against time (t).

**Figure 8:**
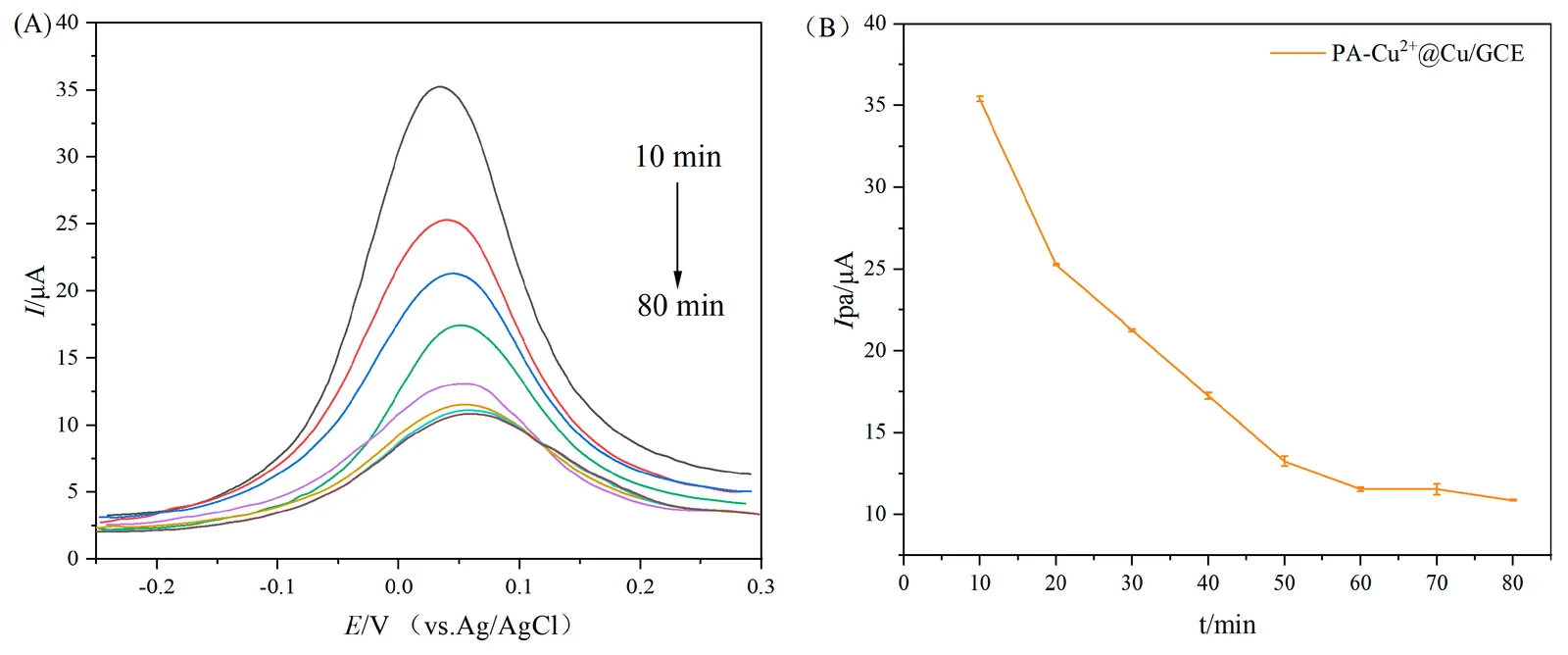
(**A**) Time-optimized SWV plot of PA-Cu^2+^@Cu/GCE and (**B**) its oxidation peak current value (Ip) plotted against time (t).

**Figure 9:**
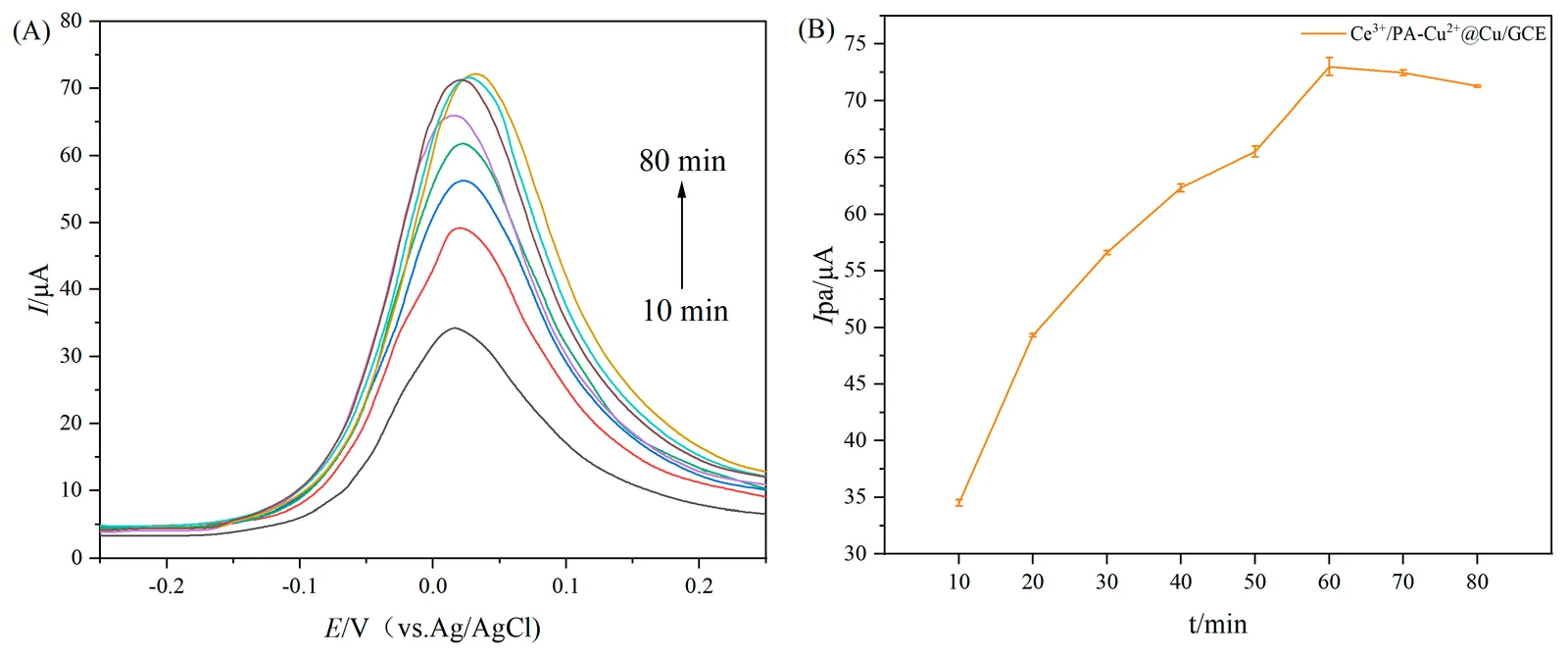
(**A**) SWV plots of Ce^3+^ composite at different times and (**B**) its oxidation peak current value (Ip) plotted against time (t).

**Figure 10:**
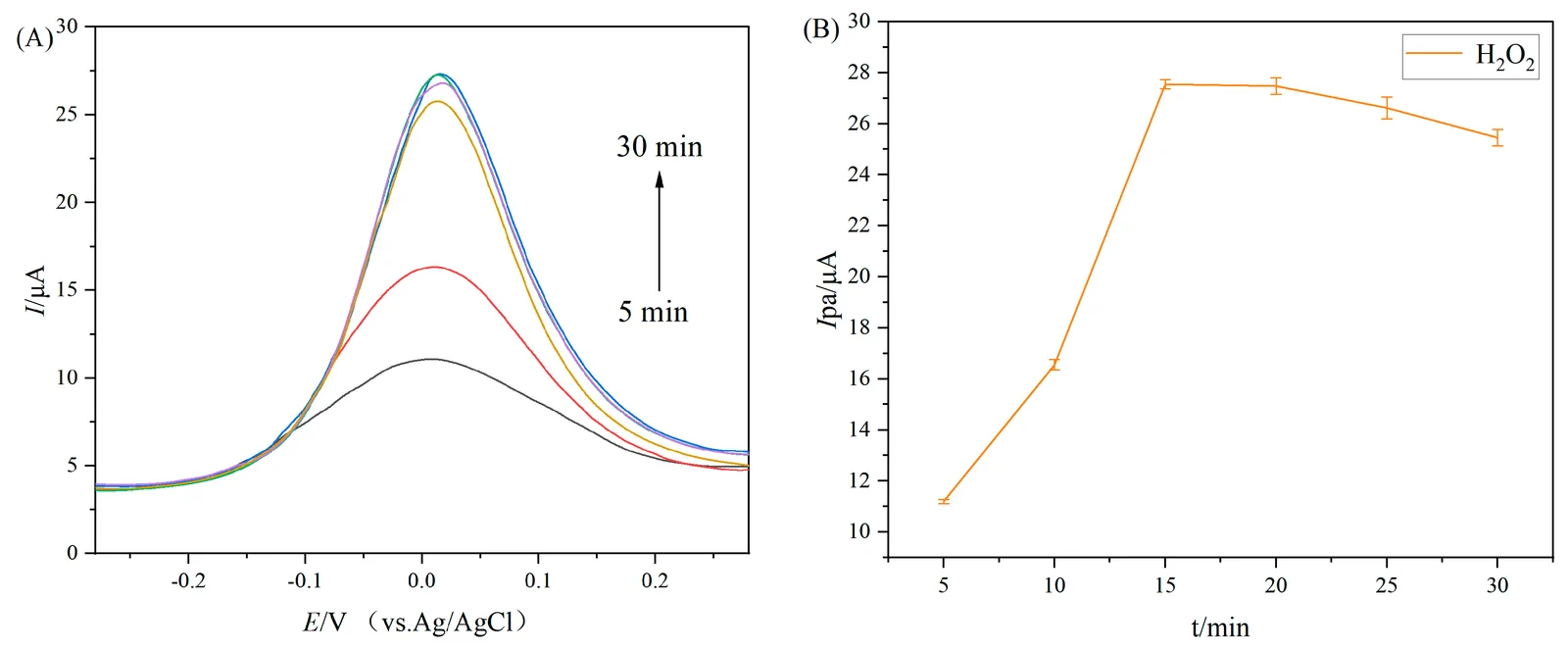
(**A**) Reaction time optimized SWV plot for H_2_O_2_ and (**B**) its oxidation peak current value (Ip) plotted against time (t).

**Figure 11:**
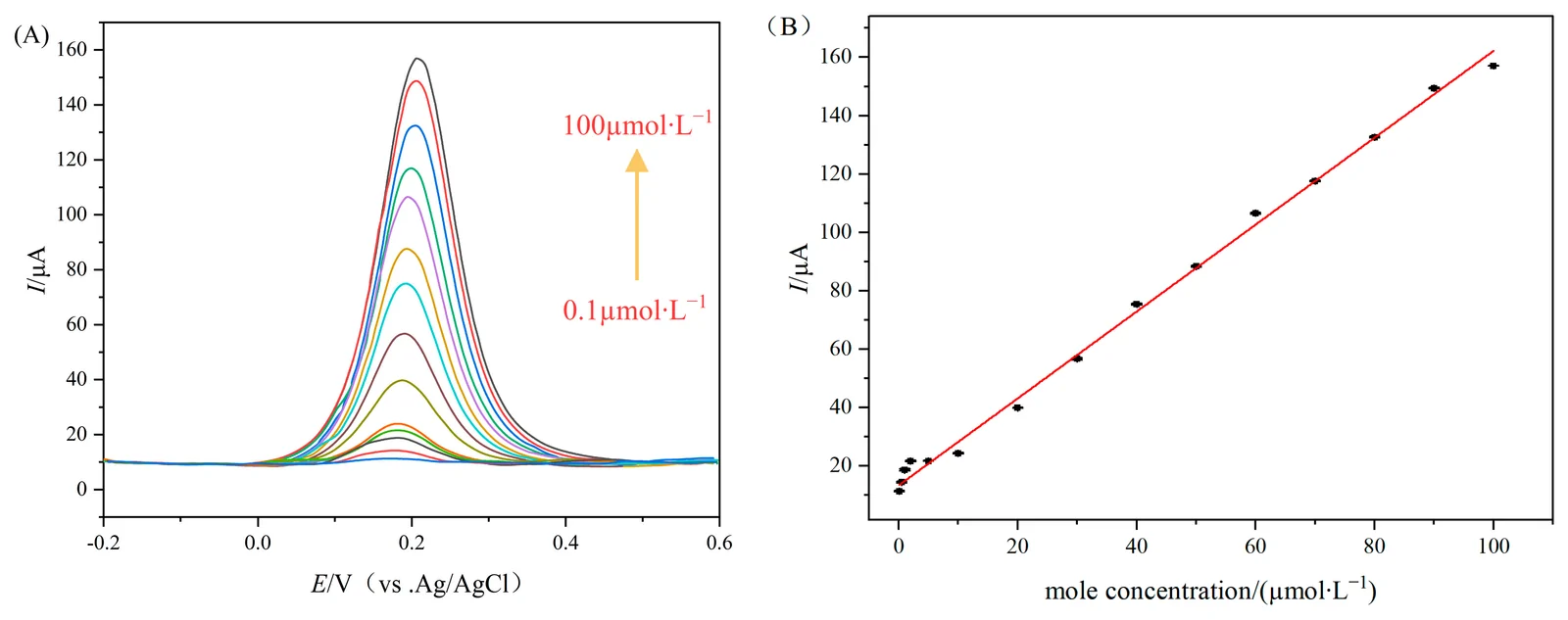
(**A**) DPV detection curves of Ce^3+^/PA-Cu^2+^@Cu/GCE for different concentrations of H_2_O_2_ and (**B**) the fitted linear relationship of IPa (peak current) for different concentrations of H_2_O_2_ being oxidized.

**Figure 12:**
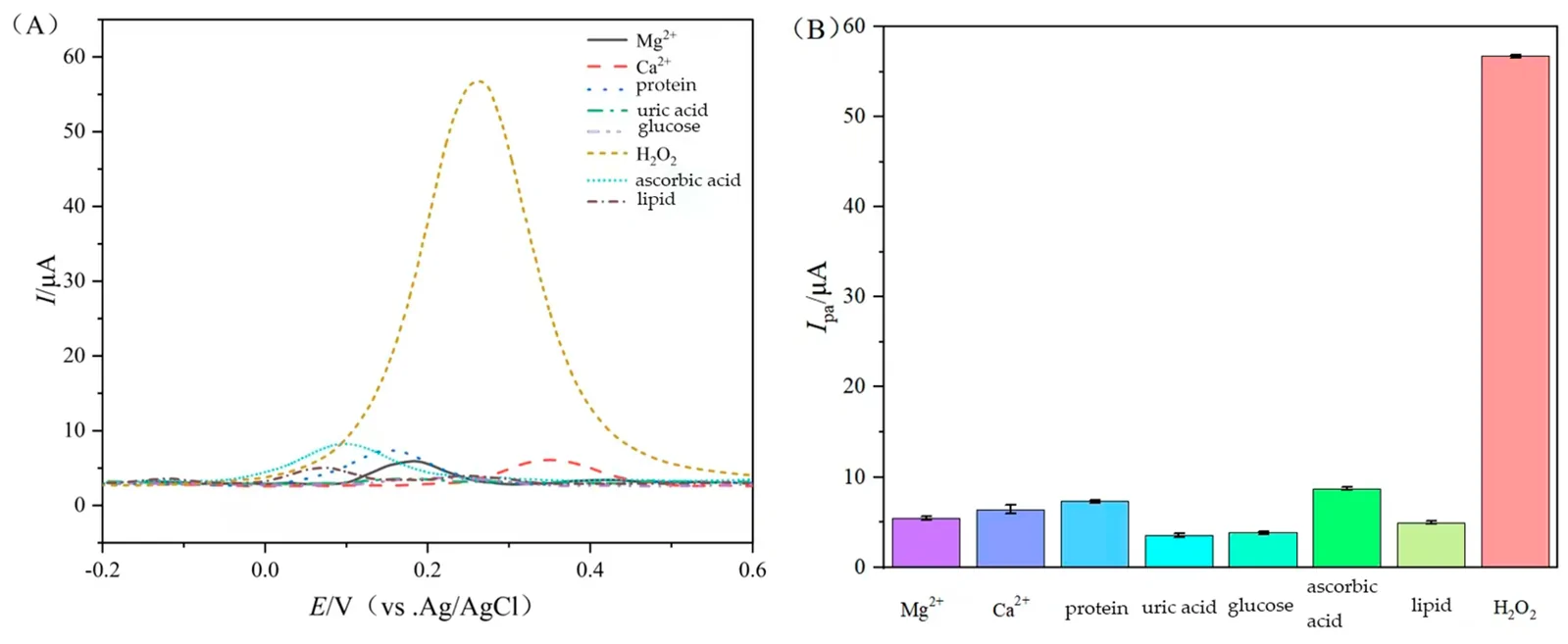
(**A**) Plot of DPV response of interferers to the sensor and (**B**) histogram of Ipa of different interfering species for corresponding conditions.

**Figure 13:**
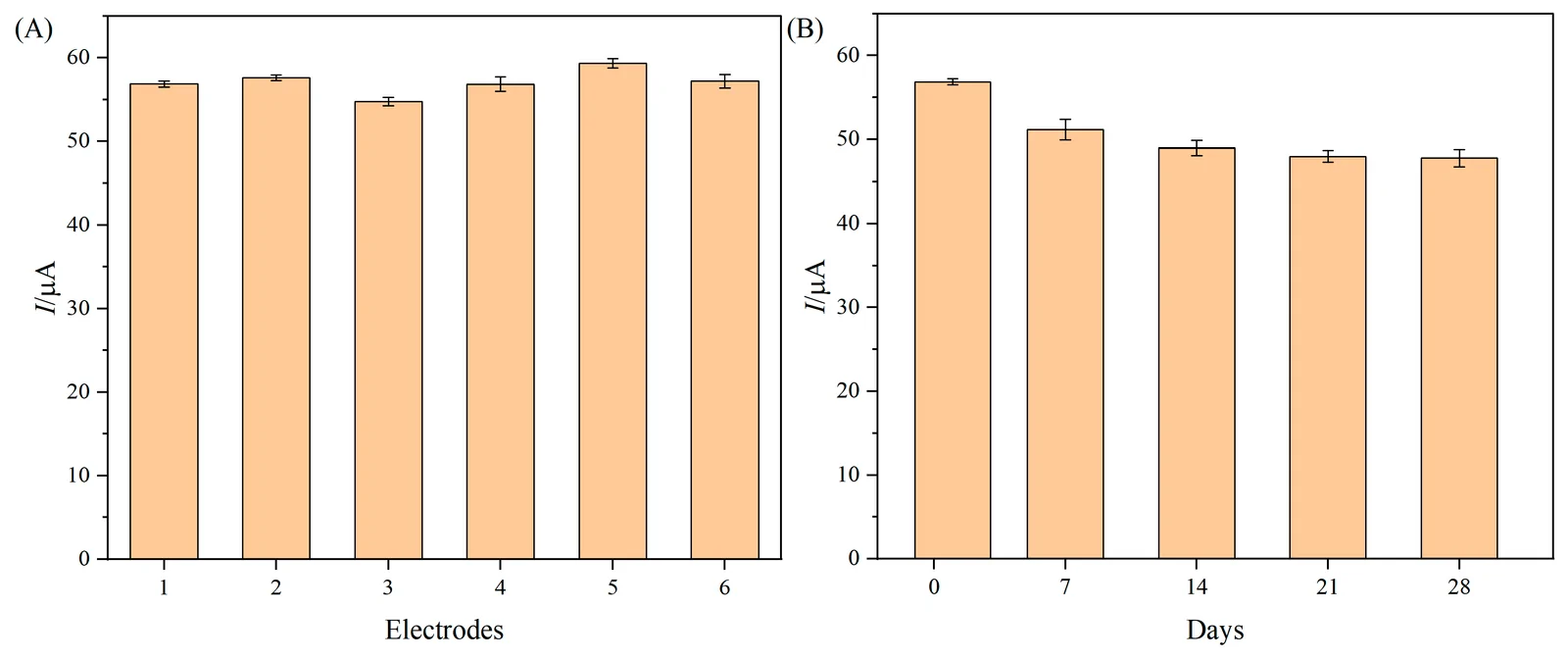
(**A**) DPV measurement of 20 μmol∙L^−1^ H_2_O_2_ by the same sensor prepared with different electrodes; and (**B**) DPV measurement of H_2_O_2_ oxidation peak currents by the modified electrodes after different days at room temperature.

## Data Availability

The original contributions presented in this study are included in the article. Further inquiries can be directed to the corresponding author(s).
